# Age-dependent integration of cortical progenitors transplanted at CSF-neurogenic niche interface

**DOI:** 10.3389/fcell.2025.1577045

**Published:** 2025-07-03

**Authors:** Gretchen Greene, Nikorn Pothayee, Jahandar Jahanipour, Hyesoo Jie, Jung-Hwa Tao-Cheng, Emily Petrus, Dragan Maric, Alan P. Koretsky

**Affiliations:** ^1^ Laboratory of Functional and Molecular Imaging, National Institute of Neurological Disorders and Stroke, National Institutes of Health, Bethesda, MD, United States; ^2^ Flow and Imaging Cytometry Core Facility, National Institute of Neurological Disorders and Stroke, National Institutes of Health, Bethesda, MD, United States; ^3^ Electron Microscopy Facility, National Institute of Neurological Disorders and Stroke, National Institutes of Health, Bethesda, MD, United States; ^4^ Department of Anatomy, Physiology and Genetics, Uniformed Services University of the Health Sciences, Bethesda, MD, United States

**Keywords:** neural precursor cell, cell transplantation, aging, graft-host connectivity, tissue regeneration

## Abstract

There has been renewed interest in neural transplantation of cells and tissues for brain repair. Recent studies have demonstrated the ability of transplanted neural precursor cells and *in vitro* grown organoids to mature and locally integrate into host brain circuitry. Most studies have focused on how the transplant behaves and functions after the procedure, but the extent to which the host brain can properly innervate the transplant, particularly in the context of aging, is largely unexplored. Here we report that transplantation of rat embryonic cortical precursor cells into the cerebrospinal fluid-subventricular zone (CSF-SVZ) interface of adult rat brains generates a brain-like tissue (BLT) at an ectopic site. This model allows for the assessment of precursor cell development, cellular interactions, and graft-host connectivity as a function of host age. We found that the transplanted precursor cells initially proliferated, then differentiated, and developed into mature BLTs, which received supportive cellular components from the host including blood vessels, microglia, astrocytes, and oligodendrocytes. There was integration of the BLT into the host brain which occurred at all ages studied, suggesting that host age does not affect the maturation and integration of the precursor cell-derived BLT. Long-range axonal projections from the BLT into the host brain were robust throughout the different aged recipients. However, long-distance innervation originating from the host brain into the BLT significantly declined with age. This work demonstrates the feasibility and utility of integrating new neural tissue structures at ectopic sites into adult brain circuits to study host-transplant interactions.

## 1 Introduction

Neurons of the central nervous system (CNS) generally lack the ability to repair and regenerate following injury or disease (Harel and Strittmatter, 2006; Varadarajan et al., 2022). Cell and tissue transplantation aimed at forming a defined brain tissue presents a promising strategy for repairing and restoring brain function in individuals affected by neurodegenerative diseases and brain injuries (Barker, Götz, and Parmar 2018; Grade and Götz, 2017). Early studies on fetal tissue transplantation demonstrated the ability of embryonic neuronal tissue to survive and form functional connections when implanted into young rodent brains (Gash, Collier, and Sladek, 1985; Girman and Golovina, 1990; Lu and Norman, 1993). Transplantation of more defined neuronal cell grafts and neuronal progenitor cells (NPCs) showed that implanted cells could differentiate into mature neurons capable of extending local and long-range projections in the brain and spinal cord (Denham et al., 2012; Falkner et al., 2016; Gaillard et al., 2007; Lu et al., 2012; Michelsen et al., 2015; Pothayee et al., 2018). In addition to efferent projections from the graft or transplant derived tissue, some studies have shown functional integration of the new tissue through the establishment of local host-transplant synapses (Espuny-Camacho et al., 2018; Falkner et al., 2016; Kumamaru et al., 2019; Michelsen et al., 2015; Palma-Tortosa et al., 2020; Pothayee et al., 2018). Recently, advances in the development of 3D neural organoids have demonstrated the ability to generate autologous complex brain tissues, and numerous studies have successfully transplanted human neural organoids into rodent brains (Jgamadze et al., 2023; Kelley et al., 2024; Kitahara et al., 2020; Mansour et al., 2018; Revah et al., 2022; Velasco et al., 2019; Wang et al., 2024). Both organoid and cell transplantations offer promising avenues for repairing neural circuits and studying the mechanisms of postnatal circuit formation.

The majority of transplantation studies have demonstrated the maturation and integration of neural transplants–either organoids or cell-derived–implanted into newborn or critical period, young host brains. It remains unclear if high level integration and bidirectional graft-host cellular interactions can occur in aged animals. For example, it is unclear whether new, long-range axonal projections which innervate the transplant from distant host brain sites can form in aged animals. We reported previously that the CSF environment enabled early embryonic neural precursor cells to grow into brain-like tissues (BLTs) without signs of teratoma formation (Pothayee et al., 2018). Extensive integration of host cells and axons projecting from the BLT into the host were observed. The ectopic site of tissue growth makes this an attractive model to study graft-host integration and new circuit formation. However, a limitation of the previous study included the nonspecific location of the BLT’s site of implantation. In this study, the BLT was grown near the subventricular zone (SVZ), close to the rostral migratory stream (RMS). The ability of transplanted cells to mature, send projections into the host brain, and importantly recruit specific, long-distance innervation from the host was investigated.

Aging negatively impacts new cell and circuit formation (Babcock et al., 2021; Couillard-Despres, Iglseder, and Aigner, 2011). Therefore, we hypothesized that an increase in host age would cause impaired survival and integration of the BLT with the host neural circuitry. Here we tested the limits of aging’s impact on these factors by studying the cellular and neuronal functional integration of the BLT with the host at three ages. Using this approach, we found that host rats aged up to 1-year at time of implantation did not limit or alter the growth potential and phenotype of the BLT or the host contributions to the BLT such as blood vessels and microglia. However, with increasing recipient age, the prevalence of long-range connections from the host brain projecting into the BLT decreased. Despite this decrease, new projections into the BLT from the host could still be detected in one-year-old host animals.

## 2 Materials and methods

### 2.1 Animal procedure

All animal procedures were handled according to the Institute of Laboratory Research guidelines and were approved by the Animal Care and Use Committee (ACUC) of the National Institute of Neurological Disorders and Stroke.

### 2.2 Cell isolation and FACs sorting

Embryos from transgenic Lewis rats expressing green fluorescent protein (GFP) were isolated at embryonic day 13.5 (E13.5) according to the approved ACUC protocol (Pothayee et al., 2018). The dorsal telencephalic region of developing cortical tissues were carefully dissected under a magnifying scope. The tissue was then dissociated using a papain-based enzyme protocol, as previously described (Maric and Barker, 2005). These cells were washed and maintained in neurobasal media prior to cell sorting. To purify the early neural stem/precursor cells, fluorescence-activated cell sorting (FACS) was used to remove lineage-committed neuronal and glial progenitors and their post-mitotic counterparts, as well as to deplete non-neural cell phenotypes (microglia, endothelial cells, pericytes) leaving the remaining lineage-negative (Lin^−^) neural stem/precursor population as previously published (Maric et al., 2007; Maric and Barker, 2005). Briefly, telencephalic cell dissociates were stained using a panel of antibodies targeting the following surface markers: CD11b (to identify microglia), CD31 (to identify endothelial cells), NG2 proteoglycan (to identify pericytes), a cocktail of antibodies targeting A2B5, CD15 and CD24 (to identify neuroglial progenitor cells), GLAST (to identify radial glial cells and differentiating astrocytes), O4 (to identify oligodendroglia progenitors and their differentiating progeny), and a cocktail of antibodies targeting CD57, PSA-NCAM and GT1b gangliosides, via binding of tetanus toxin C-fragment (TnTx) and anti-TnTx antibody, to identify neuronal progenitors and differentiating neurons. Lin^−^ cortical NSCs were then purified by applying a lineage-dumping sorting protocol excluding all cell phenotypes expressing any of the markers listed above. Finally, a uniform single cell suspension of 5 × 10^4^ Lin^−^ cells/μL (3 × 10^6^ cells in 60 μL) was prepared in neurobasal media (ThermoFisher Scientific, MA) supplemented with growth factors (bFGF and EGF, 20 ng/mL) and kept at 4°C prior to the implantation.

### 2.3 Implantation into neurogenic niches at CSF-SVZ brain tissue interface

Wild-type Lewis rats were either 3-week, 4-months, or 12-months old on the day of stereotactic implantation. The animals were anesthetized under 5% isoflurane in a 30% oxygen/70% nitrogen (oxygen-enhanced air) gas mixture. After anesthesia was induced, isoflurane was adjusted to 2%, and the pedal reflex was checked to ensure the animal did not have a pain reflex at which point the animal was moved to a stereotactic frame where isoflurane was supplied through a nose cone. Under sterile conditions, a 1 mm burr hole was drilled in the skull of the animal (+1.5–1.6 AP and +1.5–1.6 ML from bregma and 3.8 DV from skull surface for 3-week-old recipient rats) above the lateral ventricles using stereotaxic coordinates from the Paxinos and Watson rat brain atlas. The cell suspension was loaded into a 10 μL glass syringe (Hamilton, MA) equipped with a 31-gauge needle. The needle was lowered slowly and placed at a 4-mm depth from skull surface. 1μL of cell suspension containing 5 × 10^4^ Lin^−^ cells/μL was slowly levered using a hand push over a 1-min period. After injection, the needle was left in place for 3 minutes to prevent backflow before removing. The burr hole was sealed with bone wax and the skin was sutured. Immediately after surgery, the animals were given analgesic (ketoprofen, 5 mg per kg) and placed in a heated recovery chamber. The animals were monitored for any sign of complication and returned to their home cages. For older hosts, 4-month and 12-month-old rats, coordinates were adjusted accordingly. For four-month-old rats, coordinates were +1.6–1.7 AP, +1.6–1.7 ML from Bregma and 4.2 DV from skull surface. For 12-month-old rats, the coordinates were +1.7–1.8 AP, +1.7–1.8 ML and 4.4-4.5 DV from skull surface.

### 2.4 MRI

MRI was performed following implantation to monitor growth kinetics of the new tissue in the ventricle. All MRI experiments were done on an 11.7 T animal MRI (30 cm 11.7 T horizontal magnet, Magnex Scientific, Oxford, England; MRI Electronics, Bruker Biospin, Billerica, MA) with a 12-cm integrated gradient shim system (Resonance Research Inc., Billerica, MA) using a custom-built volume transmit coil and a custom built, receive-only 2-coil array surface coil. Flash 3D gradient echo sequences were used for all MRI acquisitions. For *in vivo* imaging, the following parameters were used: field of view (FOV) = 1.92 cm^3^, matrix size 256 Å∼ 256 Å∼ 256 (100 μm isotropic resolution), 12.5 kHz bandwidth, TE = 8 ms, TR = 25 ms, and flip angle = 8°.

### 2.5 Immunostaining

Rats were first deeply anesthetized under 5% isoflurane in a 30% oxygen/70% nitrogen gas mixture and transcardially perfused with PBS followed by 5% formalin/PBS. Brains were dissected from the skull following perfusion and post-fixed for 24 h in the same fixative. Brains were then placed in 15% sucrose/PBS overnight at 4°C and moved to 30% sucrose/PBS at 4°C until the tissue sank to the bottom of the solution. Brains were embedded in Optimum Cutting Temperature (OCT) medium and frozen in isopentane. For immunohistochemistry to characterize cell phenotypes, rats were perfused 8–10 weeks following cell implantation. Frozen brains were sectioned sagitally using a cryostat and 10 μm thick sections were collected on Leica Apex Superior Adhesive slides (product#3800080). 10-μm sagittal brain sections were immunoreacted for 1 h at room temperature (RT) using 1 μg per mL final concentration (diluted in PBS supplemented with 1% bovine serum albumin, PBS/BSA) of the following primary antibodies (vendor source and product number are indicated in parentheses): mouse IgG1 anti-rat endothelial cell antigen (RECA1) (Abcam, ab9774), mouse IgG1 anti-CD68/ED1 (Thermo Fisher Scientific, MA5-16654), mouse IgG2a Olig2 (EMD Millipore, MABN50), mouse IgG2b anti-proliferation cell nuclear antigen (PCNA) (Abcam, ab184660), mouse IgG3 anti-glutamic acid decarboxylase 67 (GAD67) (Santa Cruz Biotechnology, sc-28376), mouse IgG2a anti-S100 (EMD Millipore, MAB079-1), anti-myelin basic protein (MBP) (EMD Millipore, MAB386), chicken IgY anti-glial fibrillary acidic protein (GFAP) (EMD Millipore, AB5541), rabbit IgG anti-Iba1 (Wako Chemicals, 019-19741), rabbit IgG anti-Doublecortin (DCX) (Abcam, ab18723), rabbit IgG anti-guinea pig IgG anti-NeuN (EMD Millipore, ABN90P). Select combinations of immunocompatible primary antibodies (i.e., antibodies from a different host or belonging to a different immunoglobulin class or subclass) from the list above were also used for multiplexing two or more biomarkers at the same time, as described previously (Maric et al., 2021) and further detailed in the results section. The sections were then washed in PBS/BSA and immunoreacted using a 1 μg per mL of the appropriate secondary antibodies (Thermo Fisher Scientific, Li-Cor Biosciences) conjugated to one of the following spectrally compatible fluorophores: Alexa Fluor 350, Alexa Fluor 405, Alexa Fluor 430, Alexa Fluor 488, Alexa Fluor 546, Alexa Fluor 594, Alexa Fluor 647, IRDye 680LT, or IRDye 800CW. Some sections were also counterstained with 1 μg per mL DAPI (Thermo Fisher Scientific) to facilitate cell counting. The slides with labeled tissue sections were then cover slipped using Immu-Mount medium (Thermo Fisher Scientific, MI). All sections were imaged using an Axio Imager Z.2 multi-channel scanning fluorescence microscope (Carl Zeiss, Thornwood, NY) equipped with a 20X Plan-Apochromat (Phase-2) objective Carl Zeiss), a high resolution ORCA-Flash 4.0 sCMOS digital camera (Hamamatsu Photonics, Japan) sensitive to a broad-spectrum of emission wavelengths, including those approaching infrared, a 200W X-Cite 200DC broad-spectrum light excitation source (Lumen Dynamics), and 10 self-contained excitation/dichroic/emission filter sets (Semrock, Rochester, NY) optimized to detect the following fluorophores with minimal spectral crosstalk: DAPI, Alexa Fluor 350, Alexa Fluor 405, Alexa Fluor 430, Alexa Fluor 488, Alexa Fluor 546, Alexa Fluor 594, Alexa Fluor 647, IRDye 680LT, and IRDye 800CW. Each labeling reaction was sequentially captured using filtered light through an appropriate fluorescence filter set and the images individually digitized at 16-bit resolution using the ZEN imaging program (Carl Zeiss). An appropriate color table was applied to each image to either match its emission spectrum or to set a distinguishing color balance. The pseudo-colored images were then converted into TIFF files, exported to Adobe Photoshop and overlaid as individual layers to create multi-colored merged composites.

For immunohistochemistry to assess development of long-range connectivity and blood vessel density, rats were perfused 8–10 weeks following cell implantation. 30-μm thick coronal cryosections were immunostained with anti-GFP and anti-RECA-1 antibodies using a standard procedure for free-floating immunohistochemistry. Primary antibodies used for staining were mouse IgG1 anti-rat endothelial cell antigen (RECA1) (Abcam, ab9774) and chicken polyclonal anti-GFP (Abcam, ab13970). These were visualized using Alexa Fluor 594 conjugated goat anti-mouse IgG1 and Alexa Fluor 488-conjugated goat anti-chicken IgY secondary antibodies (Thermo Fisher Scientific), respectively. Sections were mounted onto slides and cover slipped using Immu-Mount and imaged with a Nikon Eclipse Ti microscope (Nikon, CA) using a ×20 objective.

### 2.6 Immunogold labeling and electron microscopy

8–10 weeks following cell implantation, rats were perfused according to the protocol described above but using 4% paraformaldehyde in PBS as fixative, and immunolabeled as described before (Tao-Cheng et al., 2021). Briefly, fixed brains were sectioned using a vibratome into 100 μm thick coronal sections and processed free-floating in 24-well cell culture plates. Samples were made permeable and blocked with 0.1% saponin and 5% normal goat serum in PBS for 40–60 min, incubated with primary and then secondary antibodies (Nanogold, at 1:200, Nanoprobes, Yaphand, NY) for 1 h, fixed with 2% glutaraldehyde in PBS for 30 min, and stored at 4°C in fixative. Samples were then silver enhanced (HQ kit, Nanoprobes), treated with 0.2% osmium tetroxide in 0.1 M phosphate buffer at pH 7.4 for 30 min on ice, then block stained with 0.25% uranyl acetate in acetate buffer at pH 5.0 for 1 h at 4°C, dehydrated in graded ethanol, and embedded in epoxy resin.

### 2.7 Slice electrophysiology

To detect monosynaptic connections between the host and transplant, the ChR2-AAV used was: AAV2/9. hSynapsin.hChR2(H134R)-EYFP.WPRE.hGH, RRID: Addgene_26973 (Penn Vector Core, University of Pennsylvania). 8 weeks after NPC implantation, ChR2-AAV was injected stereotactically targeting either the host frontal cortex or the BLT. Six weeks after ChR2-AAV injection, rats were deeply anesthetized with 5% isoflurane until the absence of righting reflex was observed. Animals were transcardially perfused with dissection buffer (80 mM NaCl, 3.5 mM KCl, 1.25 mM H2PO4, 25 mM NaHCO3, 4.5 mM MgSO4, 0.5 mM CaCl2, 10 mM glucose, and 90 mM sucrose) and the brain was removed. Tissue blocks were sectioned into 300 µm thick slices on a Leica VT1000S vibratome (Leica Biosystems Inc.). Slices remained in the dissection buffer for 1–3 h before being transferred to the submersion recording chamber continually perfused at 2 mL/min with artificial cerebral spinal fluid (ACSF; 124 mM NaCl, 5 mM KCl, 1.25 mM NaH2PO4·H2O, 26 mM NaHCO3, 10 mM dextrose, 2.5 mM CaCl2, and 1.5 mM MgCl2), which was bubbled with 95%/5% O2/CO2. The internal solution was K-gluconate based, which contained the following: 130 mM K-gluconate, 10 mM KCl, 0.2 mM EGTA, 10 mM HEPES, 4 mM MgATP, 0.5 mM NaGTP, and 10 mM Naphosphocreatine; pH 7.3, 280–290 mOsm). Cells with an access resistance higher than 25 MΩ and input resistance lower than 100 MΩ were discarded. An axon patch-clamp amplifier 700B (Molecular Devices) was used for voltage clamp recordings. Data were acquired through pClamp10 and analyzed with Clampfit 10.4 software (Molecular Devices).

Neurons in the host or the transplant tissue were visually identified for recording. ChR2 was activated using a 455-nm light emitting diode (LED) DC2100, illuminated through the ×40 objective lens and controlled by a digital stimulator (Cygnus DG4000A), both from ThorLabs. Cells which responded to LED stimulation with a short latency (<50 ms) were counted as responding monosynaptically. Neurons with no response, even to maximum LED intensity, were counted as not responding.

### 2.8 *In vivo* recording

The rats were anesthetized with urethane (1.25 g/kg, i. p. injection, Sigma-Aldrich). Once the absence of the hind-paw pinch reflexes was observed, the rat was mounted to a stereotaxic frame (Stoelting Inc.). An ocular ointment was applied to the eyes of the rat to prevent drying. Body temperature was kept constant at 37°C by using hand warmers and monitored via a thermometer throughout the entire recording sessions. Craniotomy (0.5-mm) was performed on the left orbitofrontal cortex. A 32-channel silicon probe (A1x32-6mm-50-177; NeuroNexus) was lowered to the target depth from the brain surface. For rats with BLTs, MRI scanning was performed to locate the BLTs and determine a coordinate for electrode placement. The electrode Ag/Ag-Cl pellet (EP1, World Precision Instruments) was inserted distal to the recording site as a ground and reference. Electrophysiological signals were sampled at 30 kHz (SmartBox Pro, NeuroNexus). Odor stimulation was delivered through a customized odor stimulator that used pneumatic pinch valves to provide the odor stimulus (10% amyl acetate or mineral oil) without electrical noise. A vacuum system removed excess odorant effectively for the next trial and was balanced with the incoming air. One trial consisted of 800 ms odor stimulation and 1.5 s vacuum after 6 s from odor stimulus. Five trials were conducted with a 1 min inter-trial interval. Local field potential (LFP) data were analyzed using Spikes2 software (version 8.10a, Cambridge Electronic Design Limited). LFP signals were down sampled at 1 kHz and band-pass filtered at 0.1–300 Hz. Odor stimulation evoked LFP signals were averaged and measured the LFP peak amplitude, onset time, and spectra.

### 2.9 Retrograde AAV viral tracing

8–10 weeks post-implantation, retrograde AAV-CAG-mCherry vectors (4 × 10^12^ μg/mL) were injected into the transplant tissue with MRI-guided coordinates. 250 nL of viral solution was injected at 50 nL/min using osmotic pump and waited 5 min after injection before the needle was retracted to minimize the backflow and leakage of the solution. The animals were euthanized for histological examination 2 weeks after the injection. Brains were collected and cryopreserved as described above. Frozen brains were sectioned into 30 μm thick coronal sections collected free-floating and placed in PBS. Sections were stored at 4°C until mounting on Superfrost plus microscope slides and cover slipped with ProLong Gold Antifade Mountant (ThermoFischer Scientific).

### 2.10 Image processing and quantification

To quantitatively assess the cell phenotype across the age groups, first, we manually delineated the regions of interest in both the BLT and host frontal cortex. Next, we utilized the Faster-RCNN method, as previously described, to locate cell nuclei using the DAPI and histone channels. To classify the located cells into the major cell types of neurons, astrocytes, microglia, and oligodendrocytes, we utilized our cell classification model to extract a comprehensive representation of major cell types by employing the Capsule Network method (Maric et al., 2021). This abstract representation encapsulated information related to cell shape, morphology, and other features based on specific markers (NeuN, S100B, IBA1, Olig2). With the assistance of the Napari interactive viewer (Chiu et al., 2022). We performed thresholding on this abstract representation, to identify the major cell types in both the BLT and Cortex regions. We also measured the GFP intensity within bounding boxes and applied the thresholding technique to distinguish GFP^+^ cells. Further analysis within each cell class involved a comparison between GFP^+^ and GFP^−^ cells. Additionally, for blood vessels labeled with the RECA-1 biomarker, we employed the Segment Anything Model (Kirillov et al., 2023). This segmentation process was initiated by manually acquiring image samples and generating a segmentation mask. We calculated and compared the RECA-1 density between the BLT and host Cortex regions based on the segmentation mask area percentage.

### 2.11 Statistical information

Data are expressed as mean ± standard error of the mean. Statistical analyses were performed using GraphPad (La Jolla, CA, USA). When evaluating cell percentages between the BLT and cortical regions, the comparison between the two groups was made using an unpaired T-test. Additionally, an unpaired T-test was used to compare the RECA-1 percent area between the BLT and cortical region to evaluate vascular density. When comparing parameters between the three age groups, a one-way ANOVA and Tukey’s post-hoc test for multiple comparisons was used. This was the analysis completed for the cell origin (GFP-positive and GFP-negative), anterograde projection density, and retrograde cell body density comparisons in which case all three age groups were directly compared.

## 3 Results

### 3.1 Formation and integration of neural precursor cells transplanted at the CSF-SVZ interface

**FIGURE 1:**
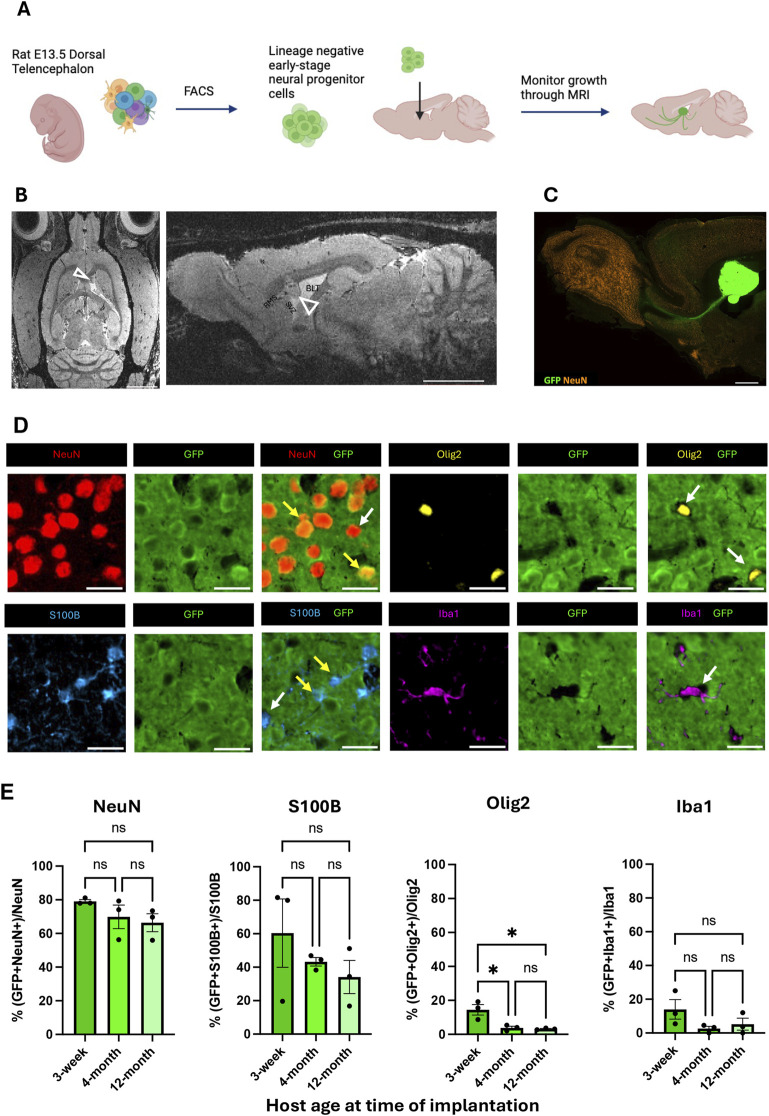
Formation and integration of BLT at the CSF-SVZ-RMS interface. **(A)** Workflow of cell implantation (created with BioRender.com). Embryonic rat cortical neural precursor cells (NPCs) were isolated from E13.5 dorsal telencephalic tissue dissociates and purified with fluorescence-activated cell sorting prior to implantation at the SVZ next to the RMS. **(B)** Representative axial and sagittal MRI of rat brain 8 weeks post-implantation (PI), at which point the precursor-generated brain-like tissue (BLT) transplants were clearly visible (open arrowheads). Scale bar = 2 mm. **(C)** Sagittal brain section showing GFP-positive BLT (green) near the SVZ/RMS injection site bordering the CSF-filled lateral ventricle (LV). Scale bar = 2 mm. **(D)** Fluorescent images from immunostaining characterizing origin of each cell type. IHC was performed from brain samples collected 8 weeks after cell implantation into 3-week, 4-month, and 12-month-old rats. At time of euthanasia, the animals were approximately 3-months, 5-months, and 14-months-old, respectively. GFP positive cells signify transplant origin (yellow arrows), whereas GFP negative cells are derived from the host (white arrows). NeuN-positive cells represent neurons. S100B-positive cells represent astrocytes, Iba1-positive cells represent microglia, and Olig2-positive cells represent oligodendrocytes. Scale bar = 20 μm **(E)** Bar graph showing percent of each cell type–neurons, astrocytes, microglia, and oligodendrocytes–derived from implanted cells (GFP-positive) compared between the three host age groups (n = 3, NS: P > 0.05, *:P ≤ 0.05, **:P ≤ 0.01). A one-way ANOVA followed by Tukey’s multiple comparisons test was used.

Freshly sorted lineage negative cortical neural precursor cells were isolated from primary GFP-expressing E13.5 rat embryonic dorsal telencephalic tissues as previously described (Maric et al., 2007; Pothayee et al., 2018) (Figure 1A; Supplementary Figure S1). These cells were injected in the SVZ adjacent to the RMS in adult rat brains after isolation. At 8 weeks PI, the transplanted cells formed a stable BLT which was detectable with T_2_-weighed MRI. Eight weeks PI also coincided with a cessation of proliferating (PCNA-positive) activity (Figure 1B; Supplementary Figure S2). Immunostaining with anti-GFP antibodies showed that the transplant extended projections from its implantation site into the host. These projections were predominantly located along the RMS extending into the olfactory bulb. In addition, GFP-positive projections from the BLT were observed in the frontal cortex, striatum, and thalamus where axons formed mature synapses with host neurons (Figure 1C; Supplementary Figures S3, S4). Although the aim of this work was to determine the impact of host age on the developmental propensity of implanted precursor cells, we also showed that NPCs implanted at the SVZ-CSF interface developed into a mature brain-like tissue that functionally integrated with the host brain circuit (Supplementary Results, Supplementary Figure S5).

Graft-host interactions involve multiple glial cell types (Talaverón et al., 2014). In our case, microglia, blood vessels, and most oligodendrocytes migrated into the BLT from the host while most neurons and astrocytes arose from the implanted precursor cells. An important question in cell and tissue transplantation is whether the age of recipient impacts the cellular organization and host-transplant interactions. Thus, we implanted early-stage NPCs into the SVZ-CSF region of rats that were 3-weeks, 4-months, or 12-months of age. The origin, whether transplant-derived or host derived, of each cell type within the BLT at 8-weeks PI was measured by co-staining of GFP with the cellular markers NeuN, S100B, IBA1, and OLIG2 (Figure 1D). A total of 23,100 cells were analyzed across all animals (Supplementary Table S1). Within the BLT, the implanted precursor cells primarily matured into neurons (GFP-positive and NeuN-positive) and secondarily into astrocytes (GFP-positive and S100B-positive). Approximately 70 percent of the neurons found in the BLT were GFP-positive and approximately 40 percent of astrocytes in the BLT were GFP-positive. Most oligodendrocytes and microglia were GFP-negative and were thus supplied by the host (Figure 1E). This agrees with previously reported data in younger rodents (Pothayee et al., 2018) and follows the known differentiation potential of the cells isolated from the early (E13.5) dorsal telencephalon, which, at that gestational stage, does not generate oligodendrocyte precursors (Su et al., 2023). If any microglia infiltrated the diencephalon at E13.5, they were presumably depleted by lineage negative selection using FACS. While we observed migration of host cells into the BLT, no BLT-derived neurons or astrocytes were found migrating to distal regions of the host.

### 3.2 Impact of host age on cellular composition of the BLT

**FIGURE 2:**
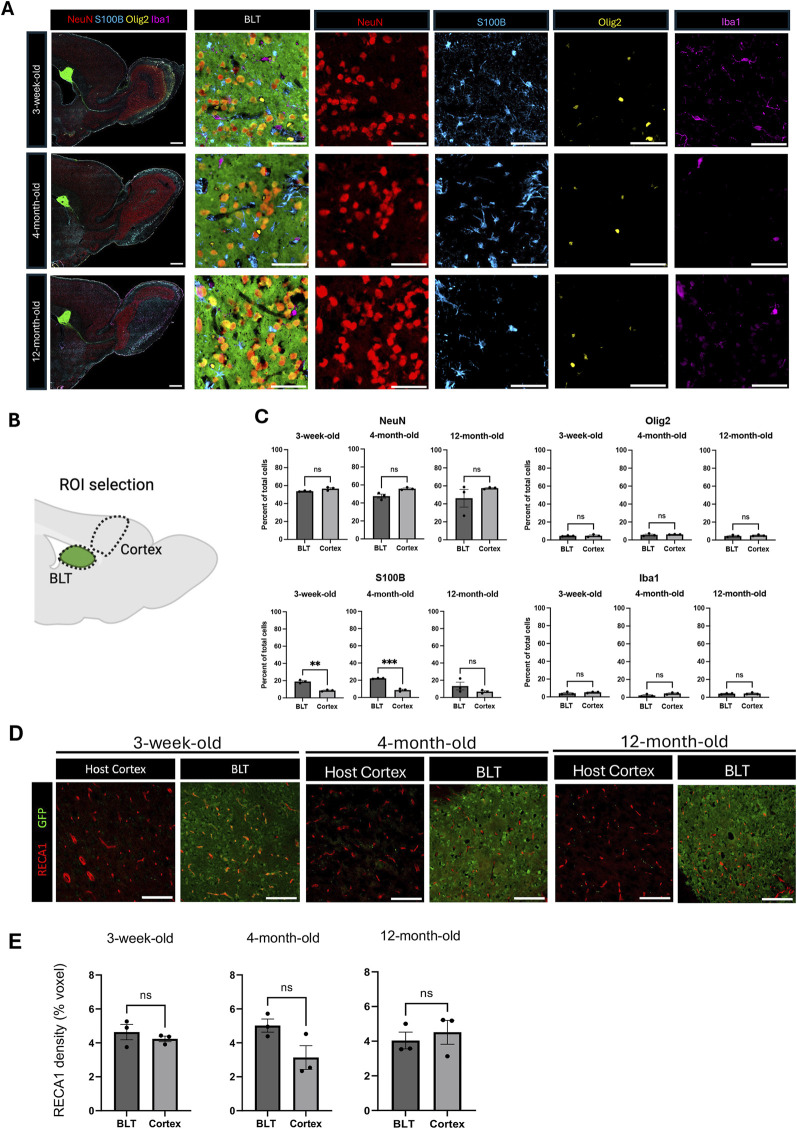
Cellular composition of the BLT is similar to the host cortex. **(A)** Representative images of sagittal brain sections from animals with BLT implants, collected 8 weeks after cell implantation into 3-week, 4-month, and 12-month-old rats. At time of euthanasia, the animals were approximately 3-months, 5-months, and fourteen-months-old, respectively. NeuN-positive cells represent neurons, S100B-positive cells represent astrocytes, Iba1-positive cells represent microglia, and Olig2-positive cells represent oligodendrocytes. Neurons, astrocytes, microglia, and oligodendrocytes were found in the transplant across the three age groups. Scale bars = 1 mm (larger field of view) and 50 μm (smaller field of view) **(B)** Diagram showing region of interest (ROI) selection for comparison of the cellular composition between the transplant and the host cortex (Created with BioRender.com). **(C)** Comparison of cellular composition of the BLT and host cortex based on the percentage of each cell type relative to the total cells in the defined ROI (n = 3). Normality was evaluated and then an unpaired T-test was used to compare the two conditions. NS means no statistical difference was observed P ≤ 0.001, *: P ≤ 0.0001. **(D)** Immunostaining with RECA-1 for identification of blood vessels. Scale bar = 100 μm **(E)** Vasculature density in the transplant and host cortex across the host ages (n = 3, NS P > 0.05). Normality was evaluated and then an unpaired T-test was used to compare the two conditions.

The BLT was comprised of neurons, astrocytes, oligodendrocytes, and microglia regardless of the age of the recipient (Figure 2A). However, it was unclear whether the overall cellular composition of the BLT derived from cortical precursors would mirror that of the adjacent host cortex. To investigate this, 8 weeks following implantation, host rats were euthanized, and the cellular composition of the BLT was evaluated using multiplexed immunohistochemistry followed by implementation of an automated cell counting algorithm. Using NeuN to label neurons, Olig2 for oligodendrocytes, Iba1 for microglia, and S100B for astrocytes, we compared the overall cellular composition of the BLT to normal frontal cortical tissue located distal from the cell implantation site (Figure 2B). Approximately 17,000 cells from the BLT and 16,000 cells from host cortex were analyzed (Supplementary Table S2). No major difference between the cellular composition of the BLT and host cortex was found, irrespective of transplant recipient host age. The percentage of neurons, oligodendrocytes, and microglia relative to the total cells in the defined region was similar between the BLT and the adjacent cortex (Figure 2C). Interestingly, the percent of total astrocytes was significantly higher in the BLT compared to the host frontal cortex in the 3-week-old (19% in BLT vs. 8.6% in cortex, p value = 0.0010) and 4-month-old group (22.17% in BLT and 8.7% in cortex, p value = 0.0001), but not significantly different in the 12-month-old group (p value = 0.2319). These findings imply that there was increased astrocyte number in the BLT relative to the cortex in younger hosts. In all hosts, the BLT was well vascularized as demonstrated by immunostaining for Rat Endothelial Cell Antigen (RECA)-1 (Figure 2D). The vessels were host derived, and angiogenesis occurred in the BLT despite the advanced age of the host. Blood vessel density was calculated based on percentage of image voxels positive for RECA-1 out of the total area. There was no significant difference between the density of blood vessels in the BLT in comparison to the host cortex in any of the age groups (three-week-old: p value = 0.4542, four-month-old: p-value = 0.6035, 12-month-old: p-value = 0.0779) (Figure 2E). Further evaluation of markers, although outside the scope of this work, such as myelination (MBP), inhibitory neurons (GAD67), and the formation of a host ependymal layer at the interface of BLT-to-CSF were observed across all age groups as well (Supplementary Figures S6-S8).

### 3.3 Long-range connectivity from BLT into host as function of age

**FIGURE 3:**
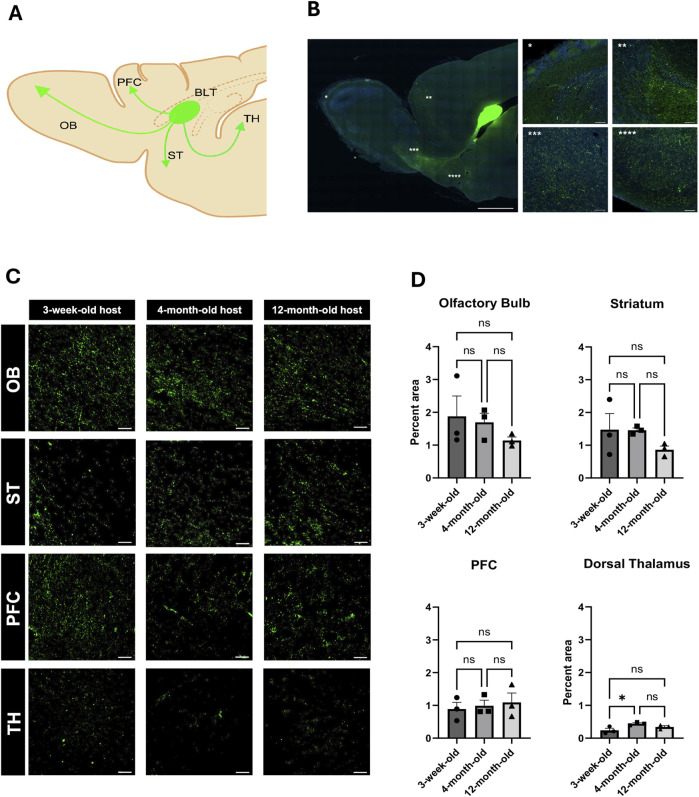
Impact of host age on innervation from BLT into host brain. **(A)** General diagram of BLT projections into different host brain regions including the olfactory blub (OB), striatum (ST), medial prefrontal cortex (PFC), and thalamus (TH) (Created with BioRender.com). **(B)** Reprisentative sagittal section of afferent axonal projections from the BLT. **(C)** High magnification of GFP-positive puncta in each area of projected afferent axonal innervation from the BLT into the host OB, ST, PFC, and TH in all recipient age groups. **(D)** Quantification based on the percent voxel of the fluorescent puncta out of total area compared across the three age groups (n = 3, NS: P > 0.05, *:P ≤ 0.05). Analysis was completed with a one-way ANOVA and Tukey’s multiple comparisons test.

Long-range connectivity between neural transplants of both cells and organoids is of great interest in order to establish functional circuits that are integrated into the host brain. Here, we sought to determine whether recipient age impacts the ability of the transplant and host to establish such connections. BLT neurons project into the host circuitry extending into the olfactory bulb, medial prefrontal cortex, and the striatum (Figures 3A,B). The percentage of GFP fluorescent puncta was highest in the olfactory bulb and striatum with densities (i.e., total pixel area) of approximately 2% in these regions and less in the prefrontal cortex with a density of about 1%. Fluorescent puncta were also noted in the thalamus and piriform cortex (Figures 3B,C, Extended Supplementary Figure 8). There was no significant difference of transplant innervation from the BLT into the olfactory bulb, striatum, and PFC across the three host age groups (Figure 3D; olfactory bulb: p value = 0.4428, striatum p value = 0.3116, PFC: p value = 0.8179). There was a significant difference seen with BLT innervation to the dorsal thalamus between the 3-week and four-month-old groups (p value = 0.0476) with an increase in innervation in the four-month-old host. There was no difference seen in thalamic innervation between the 3-week and 12-month (p value = 0.3138) or the 4-month and 12-month hosts (p-value = 0.3545). Therefore, the increase in innervation seen in the dorsal thalamus region between the 3-week and 4-month host might be due to the overall small percent area occupied by the fluorescent puncta and not reflect a true biological change in BLT innervation between the two host ages. Remarkably, the transplanted neurons are effective at sending projections into all host age groups.

### 3.4 Aging alters host brain ability to project into the BLT

**FIGURE 4:**
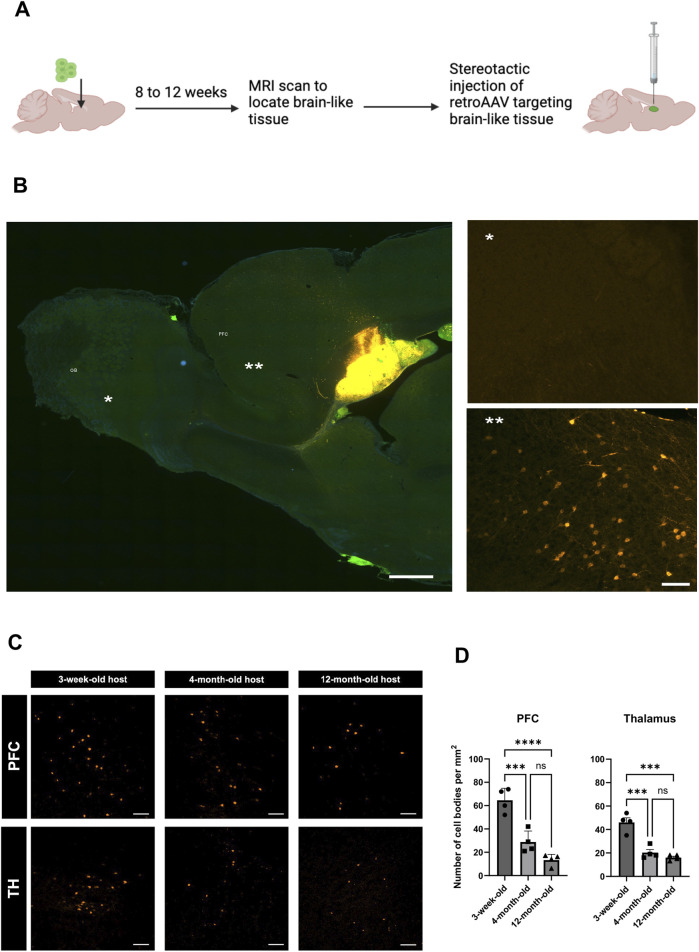
Impact of host age on innervation from host into BLT. **(A)** Experimental diagram for retrograde AAV tracing from the BLT. Cell bodies of host neurons labeled by retroAAV-mCherry (Orange). **(B)** Representative sagittal section of retroAAV-mcherry injection into the BLT and subsequent neuronal labeling. Retrogradely labeled cell bodies were mainly found in the frontal cortex of the host (**) but not in the olfactory bulb (*). **(C)** Retrograde tracer distribution in the prefrontal cortex (PFC) and thalamus (TH) across the three host ages–3-weeks, 4-months, and 12-months at time of cell implantation and 11-weeks, 6-months, and 14-months at time of retroAAV tracer injection. Representative region of interest (ROI) images showing cell bodies (orange) that were labeled with retroAAV-mcherry collected at 2 weeks post tracer injection. Scale bar = 100 μm. **(D)** Quantification of number of cells retrogradely labeled in each ROI compared between the three recipient age groups (n = 4, ***P ≤ 0.001, ****P ≤ 0.0001). A one-way ANOVA with a Tukey’s multiple comparisons test was used for the comparison.

Next, we assessed the long-distance input originating from the host brain and projecting into the transplant using retrograde viral tracing (Figure 4A) After injecting mCherry-expressing retrograde AAV into the BLT, we found labeled neuronal cell bodies located in the prefrontal cortex (PFC) and thalamus (TH) (Figure 4B) indicating that the new tissue received input from the host brain. No retrograde tracing was found in the OB of the host implying that connection with the OB was not bi-directional.

To investigated whether the host age alter connections originated from the host which projected into the BLT, we implanted the cells into 3-week-old, 4-month-old and 12-month-old hosts and performed retrograde tracing using retrograde AAV injected into the BLT within these animals. Although, host area that primarily send projection into the BLT is PFC, we also observed retrograde labeling into host thalamus (TH) (Figure 4C). We found that there was a progressive decline in the number of neurons that projected into the transplant as the host age increased. In PFC: 3-week vs. four-month p-value = 0.0006, 3-week vs. 12-month p-value <0.0001. Similarly to the PFC, projections from host TH also showed marked decline from three-week-old vs. four-month-old host (p-value = 0.0003) and three-week-old vs. 12-month-old hosts (p-value <0.0001). No retrograde labeling was observed in the host olfactory bulb, striatum, and piriform cortex at any host age. These results showed that there was a reduction of BLT innervation by the host neurons which was more robust with increasing age. Comparing the three-week-old host to four-month-old host, there was approximately a 55% decline of BLT innervation by the host neurons, and with a 12-month-old host, an almost 70% reduction in these numbers was identified (Figure 4D).

## 4 Discussion

There is renewed interest in tissue and cell implants into the central nervous system in part due to recent advances in brain organoids and the ability to differentiate patient-derived induced pluripotent stem cells into specific neuronal cells for transplantation (Jgamadze et al., 2023; Quezada et al., 2023; Velasco et al., 2019). Most studies to date have focused on the ability of the implant to survive, mature, and integrate into local circuits following transplantation (Dulin et al., 2018; Espuny-Camacho et al., 2018; Falkner et al., 2016; Gaillard et al., 2007; Gronning Hansen et al., 2020). Few studies have evaluated if and how the host brain adapts to accept the transplant. Recently, Paterno et al. investigated how the host brain impacts survival and maturation of transplanted medial ganglionic eminence (MGE) progenitors (Paterno et al., 2024). This showed that the age of the host influenced MGE progenitor cell maturation, but did not investigate specifically the host brain circuitry response to the implanted cells.

In previous work we showed that cortical precursor cells transplanted at the CSF- tissue interface can develop into a brain-like tissue with extensive interactions with the existing host brain tissue (Pothayee et al., 2018). In the present study, we demonstrate the specific source of the host’s long-range connections with the BLT. By growing the BLT at an ectopic site at the CSF-tissue interface, this approach allows for the ability to quantitate host-BLT interactions and reduce local neural interactions between the host and graft. Based on this principle, our aim was to determine how the age of the host brain environment affects maturation, integration, and further interactions between the cortical precursor cells and the host brain circuitry.

### 4.1 Cellular composition of the BLT and host contribution to BLT are not affected by host age

Extensive studies have been done on transplanting cells, fetal tissue, and organoids into different parts of the brain and spinal cord in either intact or damaged conditions (Espuny-Camacho et al., 2013; Falkner et al., 2016; Gaillard et al., 2007; Girman and Golovina, 1990; Kitahara et al., 2020; Lu et al., 2012). In this study, we implanted neural precursor cells at the CSF-SVZ brain interface near the RMS. This location is known for its ability to generate new neurons throughout life, thus offering a permissive environment for neural precursor cell survival, proliferation, and differentiation (Fuchs, Tumbar, and Guasch, 2004). This differs from our previous report in which these cells were injected directly into the CSF in the lateral ventricle and formed a BLT inside the ventricle cavity. Though they productively incorporated with the host brain tissue in this earlier study, there was less control over where that occurred and multiple tissues were formed in the CSF (Pothayee et al., 2018). Nonetheless, the earlier study showed extensive projections from the transplant to the host and some host innervation of the BLT. The growth of cells injected close to the SVZ as done in the present study followed the same pattern of proliferation and differentiation previously observed with injection to the CSF. The proliferation and differentiation of implanted cells progressed for about 8 weeks post-injection, at which point the transplant-derived BLT no longer increased in size. These findings suggest that despite using a different injection site, the implanted neural precursor cells maintained the same proliferation and differentiation potential as previously observed.

The present study evaluated how recipient age impacts the ability of the implanted precursor cells to mature and form a tissue integrated with the host brain. Very few studies to date have evaluated how aging might impact the ability of transplanted cells, organoids, or tissues to survive and mature. This question is particularly relevant given the goal of translating tissue transplantation to patients. Neurodegeneration, stroke, and other brain injuries primarily occur in adults, and the number of patients impacted increases with age (Feigin et al., 2020). Some organoid implantation studies have focused on probing human neurodevelopment (Revah et al., 2022) and, therefore, implantation occurs in neonatal hosts where the young brain environment might allow for high implant survival (Kelley et al., 2024). This is useful for studying development, but for cell replacement therapies in conditions like stroke or traumatic brain injury which often effect adults, understanding how the aged brain environment impacts neural precursor cell survival and maturation is crucial. Recently, it was shown that the maturation to different interneuron subtypes following MGE progenitor cell implantation was significantly impacted in adult host mice in comparison to neonatal and juvenile mice (Paterno et al., 2024). The aged host brain environment had not been studied in the context of cortical progenitor cell implantation.

In three-week-old, four-month-old, and twelve-month-old host rats, we observed that implanted NPCs can form a complex brain-like tissue closely resembling the cellular composition of the host cortex. The relative abundance of neurons and glia cells in the BLT was similar to the proportions seen in the normal cortex. While we observed increased astrocyte numbers in the BLT compared to the young host cortex, the values in older animals were not significantly different. This increase could be because the young host brain environment promotes astrocyte differentiation (Akdemir et al., 2020). Indeed, astrocyte ratio in the cortex of the young host studied is relatively lower compared with the older hosts.

Regardless of the age of the recipient, the host was able to vascularize the graft and supply microglia and oligodendrocytes. In the BLT formed in the 4-month and 12-month-old hosts, a higher percentage of oligodendrocytes were supplied by the host in comparison to BLTs in the three-week-old hosts. This could be due to the developmental timing of oligodendrocyte precursor cell maturation and myelination in the adult brain. Oligodendrocyte differentiation and myelination occur postnatally and continue into adulthood (Nishiyama et al., 2021). The developmental timescale of oligodendrocytes could facilitate more migration of these cells in the older host animals in comparison to the young, three-week-old host. Additionally, an ependymal lining formed along the interface of the graft and CSF in all age groups, further demonstrating the seamless incorporation of the transplant derived tissue with the host. Overall, the ability of the transplanted NPCs to form a tissue structure closely resembling the host brain demonstrates the feasibility of cell transplantation even in aged host environments.

### 4.2 The BLT sends and receives functional connections

Functional and reciprocal connections were observed between the BLT and the host brain. Axons extended from the BLT into the host forebrain and olfactory system as well as into the thalamus and adjacent striatum. While the mechanisms regulating the path by which the transplant derived BLT extended projections into the host brain are not yet characterized, it is possible that the existing host brain tissue provided guidance cues for axonal growth from the newly formed BLT into the existing brain neuronal circuitry. For example, projections into the olfactory bulb from the BLT tended to follow the nearby rostral migratory stream. However, it is not clear why the BLT sent projections to thalamus or frontal cortex especially in the older animals. The proximity of the striatum to the BLT could have facilitated projections to this region. Efferent projections from the BLT into the host did not change in most of the regions we evaluated, though there was a small but significant increase in axonal innervation into the dorsal thalamus when comparing the 3-week to the four-month-old host. The overall innervation to this region was quite small, but this change might indicate a region-specific innervation pattern. Future studies could quantitatively evaluate axonal extension from the implant using neuronal tracing injected into target regions in the host brain.

In previous work (Pothayee et al., 2018), there was evidence of the host extending axons into the BLT, but the origin of these host connections was not evaluated. Using retrograde AAV tracing, we showed that the host brain innervated the BLT mostly from the medial prefrontal cortex in the forebrain region and to a lesser extent the thalamus. Despite extensive innervation from the BLT into the host olfactory system, no reciprocal connections originating from the olfactory bulb or olfactory cortex were observed. It is believed that all long-distance connections have formed in the rodent by two to 3 weeks after birth (Price et al., 2006). Therefore, it will be important to determine how these projections from the host into the BLT form. It could be that these connections are due to axons shifting from their normal projections into the BLT, or that the BLT induces bifurcation of existing projections, least likely is the possibility that the BLT induces new axons from the host to innervate this newly formed tissue. During rodent development, for example, when the thalamo-cortical connections are made in somatosensory cortex, the thalamic connections extend to the cortex in coordination with the reciprocal cortical connections (Molnár and Blakemore, 1995). Therefore, it seems likely that the BLT extended axons into brain areas in coordination with projections that were sent into the BLT from the host. The difference in maturation and plasticity between different brain regions could determine where the BLT projects and which regions project back into the BLT. For example, the frontal cortex is known to have a later critical period then olfactory bulb. However, the observation that the host projected into the BLT even in 1 year old animals indicated that the BLT was able to recruit projections even from older host animals.

The observation of long-distance connections from the BLT into the host is consistent with previous transplant studies which have shown that these projections typically form in young host brains. Here we show that age does not impair this ability to form connections. Remarkably, increasing host age up to 1 year of age did not affect the ability of the BLT to send projections to the host. These data suggest that the BLT maintains the ability to develop and extend neural outgrowths despite an aged host environment.

### 4.3 Importance of host age on long-distance innervation from the host into transplants

There are many examples of neural transplants including cells, fetal tissue, and human cerebral organoids extending long distance neural processes into the host brain. Transplanted cells have been shown to functionally integrate into host circuitry (Dulin et al., 2018; Espuny-Camacho et al., 2018; Falkner et al., 2016; Kumamaru et al., 2019; Michelsen et al., 2015; Palma-Tortosa et al., 2020). There has been less work determining whether the host innervates the transplant over long distances. Some exceptions have shown longer distance connectivity from the host to the transplant, for example, in the spinal cord (Lu et al., 2012). Recently, numerous studies have successfully demonstrated the ability of human neural organoids to survive and mature after implantation into host rodent brains between neonatal to three-month-old hosts (Jgamadze et al., 2023; Mansour et al., 2018; Revah et al., 2022). However, most studies have not determined whether connectivity arises from local connections with host tissue on the border of the transplant versus from a long-distance projection. The impact of host age on connectivity between the human-neural organoids or cells and the rodent host brains remains unclear. Indeed, to the best of our knowledge, 6-month-old mice are the oldest host animal used for cell implantation where subsequent cell maturation has been shown (Gaillard et al., 2007).

In this study, we sought to address this issue by placing the transplant ectopically to the host brain tissue, thus minimizing innervation from existing local connections. Using this approach, we have shown that increasing host age correlated with reduced long-distance projections from the host frontal cortex and thalamus into the transplant-derived BLT, with the most pronounced reductions in 12-month-old host rats. However, the presence of minimal but detectible projections implies that making these connections is feasible. Many factors in the aged brain might impact the ability of the host to innervate the new tissue including reduced plasticity and increased neuroinflammation (Ownby, 2010; Li et al., 2023; Burke and Barnes, 2006). Future studies probing specific aspects of the aged environment following transplantation could help elucidate why a drop-off in host innervation is observed in older hosts. If the decline in bidirectional connections could be rescued, the results could have major translational implications for CNS regenerative medicine in conditions like stroke or TBI.

### 4.4 Limitation and technical considerations of the study

Attempts were made to control the location of the transplanted cells, though it is still relatively difficult to ensure that the implanted cells do not spread throughout the CSF. In some cases, when this happened, we did not observe detectable tissue located near the injection site. Possible ways to ameliorate this issue would be through the implantation of preformed tissues or cells embedded in a 3D matrix. In addition, it is unknown if human neural organoids could be successfully implanted and grown in the SVZ-CSF rat host brain with similar results to matched homotopic NPC-host species. While we show that aging negatively impacts the ability of the host brain to form long-distance connections with the NPC-derived BLT, it’s not clear whether advanced aging (e.g., 18–24 months old rats or mice) would still be permissive for generation of new long-distance connections from the BLT into the host brain and vice versa. In addition, this present study utilized transplanted cells from an early developmental stage which likely facilitates a robust host response and a greater incorporation of the transplant to the host circuitry. Additionally, rat cortical precursor cells were transplanted rather than cells of human origin due to the immunosuppression required when using a xenograft method. This strategy allowed for comparison of host-transplant interactions and does not consider the differences in the developmental timelines between human and rodent nervous systems. Future studies transplanting human-derived tissues or organoids into various host rat ages would further elucidate the importance of host age on bi-directional connectivity between various combinations of host and transplant species and ages.

## 5 Conclusion

Early cortical neural precursor cells grow, mature, and develop into a brain-like tissue after implantation at the CSF-SVZ brain interface in rodents of up to 1 year of age at time of implantation. The newly formed BLT interacts with the host brain by extending long-distance neural processes into various host brain regions including the olfactory system, medial prefrontal cortex, and the striatum. The host brain structurally incorporates the BLT by supplying it with important support cells including astrocytes, oligodendrocytes, microglia, and blood vasculature. The host also provides innervation input from the forebrain, thalamus, and striatum. Host age of up to 1 year does not affect growth and development of the BLT or any of the cellular components supplied by the host. Furthermore, the BLT projects into the host independent of age. As the age of the recipient at time of implantation increased, the long-distance host-to-graft innervation progressively decreased. With the rise of organoid brain transplantation to establish functional connectivity and modeling disease progression particularly at circuit level (Jgamadze et al., 2023; Revah et al., 2022), the influence of host age should become an important consideration. The ectopic placement of the transplant at the CSF tissue interface may enable easier assay of some host-transplant interactions. Finally, MRI was used to assess BLT growth, but it is likely that the use of MRI will grow in importance to assess functional integration and connectivity in cell and tissue transplantation studies non-invasively.

## Data Availability

The data presented in the study are deposited in FigShare, https://doi.org/10.6084/m9.figshare.29286071.
