# Supplementary material for: Age-dependent integration of cortical progenitors transplanted at CSF-neurogenic niche interface
Source: Front Cell Dev Biol. 2025 Jul 3;13:1577045. doi: 10.3389/fcell.2025.1577045 (PMC12267260; doi:10.3389/fcell.2025.1577045)
Supplement: Supplementary file 1 [file DataSheet2.pdf]

Supplementary Results

Slice electrophysiology was used to examine whether the connections between the neural transplant and the host were functional. An adeno-associated virus encoding for ChR2 expressing a yellow, fluorescent marker (AAV-ChR2-eYFP) was injected into the BLT approximately eight weeks after implantation. Six weeks after AAV transfection host neurons in the olfactory bulb area and medial frontal cortex were patched for electrophysiological recordings. Light was used to activate ChR2-expressing synapses from the transplant and monosynaptic connections were detected in both areas. Between 50-100% of host neurons in areas that were innervated by the BLT responded to light stimulation (Supplementary Figure 5A). To determine if the projections from the frontal cortex were active in the BLT, ChR2-AAV was injected into the host frontal cortex and neurons in the transplant were patched for recordings. We found that 50% of the cells in the BLT responded to light stimulation of host-innervated regions (Supplementary Figure 5B). These results demonstrate that bidirectional synaptic activity between the host and the transplant were functional. Finally, we assessed whether the neural transplant responded to external stimuli in vivo. We used odor stimulation because anterograde and retrograde tracing detected BLT integration into the olfactory system and frontal cortical regions that are involved in odor processing (Supplementary Figure 5C). A pulse of 10% amyl acetate scented air elicited robust local field potential (LFP) response, approximately 200 μV and with onset time of approximately 500 ms (N=4). The LFP response from recordings at the prefrontal cortex of the host rat brain were used a control (N=3). In prefrontal cortex, peak amplitude of LFP was approximately 300 μV and onset time was approximately 200 ms. Although, not statistically significant, LFP response from the BLT appeared to lag those from the host prefrontal cortex. Future studies can be designed to evaluate whether the BLT impacts tasks related to odor detection or discrimination.
