# Supplementary material for: Age-dependent integration of cortical progenitors transplanted at CSF-neurogenic niche interface
Source: Front Cell Dev Biol. 2025 Jul 3;13:1577045. doi: 10.3389/fcell.2025.1577045 (PMC12267260; doi:10.3389/fcell.2025.1577045)
Supplement: Supplementary file 2 [file Table1.docx]

| Marker | **NeuN** | | **S100B** | | **Olig2** | | **Iba1** | | **Total cell** | |
| --- | --- | --- | --- | --- | --- | --- | --- | --- | --- | --- |
|  | **GFP+** | **GFP-** | **GFP +** | **GFP-** | **GFP+** | **GFP-** | **GFP+** | **GFP-** | **GFP+** | **GPF-** |
| 3-week-old host_Rat1 | 1439 | 334 | 515 | 116 | 29 | 122 | 13 | 100 | 3325 | 1439 |
| 3-week-old host_Rat2 | 984 | 277 | 386 | 97 | 18 | 98 | 4 | 69 | 2321 | 984 |
| 3-week-old host_Rat3 | 237 | 67 | 19 | 78 | 2 | 21 | 8 | 24 | 569 | 237 |
| 4-month-old host_Rat1 | 501 | 175 | 165 | 187 | 6 | 106 | 0 | 25 | 1559 | 501 |
| 4-month-old host_Rat2 | 780 | 204 | 192 | 240 | 4 | 96 | 1 | 21 | 1932 | 780 |
| 4-month-old host_Rat3 | 891 | 693 | 271 | 436 | 3 | 145 | 4 | 116 | 3279 | 891 |
| 12-month-old host_Rat1 | 485 | 175 | 69 | 66 | 2 | 52 | 2 | 52 | 1245 | 485 |
| 12-month-old host_Rat2 | 106 | 46 | 21 | 104 | 1 | 31 | 3 | 22 | 567 | 106 |
| 12-month-old host_Rat3 | 712 | 561 | 52 | 99 | 2 | 71 | 0 | 72 | 2168 | 712 |

**Supplementary table 1.** Total cells analyzed for origin of cellular contribution from either BLT (GFP+) or Host (GFP-). N = 3 rats for each group.
