# Supplementary material for: Age-dependent integration of cortical progenitors transplanted at CSF-neurogenic niche interface
Source: Front Cell Dev Biol. 2025 Jul 3;13:1577045. doi: 10.3389/fcell.2025.1577045 (PMC12267260; doi:10.3389/fcell.2025.1577045)
Supplement: Supplementary file 3 [file Table2.docx]

| Marker | **NeuN** | | **S100B** | | **Olig2** | | **Iba1** | | **Total cell** | |
| --- | --- | --- | --- | --- | --- | --- | --- | --- | --- | --- |
| Region | BLT | Host Cortex | BLT | Host Cortex | BLT | Host Cortex | BLT | Host Cortex | BLT | Host Cortex |
| 3-week-old host_Rat1 | 1773 | 1342 | 631 | 217 | 151 | 80 | 113 | 126 | 3325 | 2308 |
| 3-week-old host_Rat2 | 1261 | 941 | 483 | 131 | 116 | 78 | 73 | 96 | 2321 | 1729 |
| 3-week-old host_Rat3 | 304 | 1028 | 97 | 157 | 23 | 111 | 32 | 77 | 569 | 1799 |
| 4-month-old host_Rat1 | 676 | 788 | 352 | 99 | 112 | 87 | 25 | 75 | 1559 | 1419 |
| 4-month-old host_Rat2 | 984 | 1321 | 432 | 206 | 100 | 136 | 22 | 76 | 1932 | 2303 |
| 4-month-old host_Rat3 | 1584 | 713 | 707 | 131 | 148 | 83 | 120 | 59 | 3279 | 1306 |
| 12-month-old host_Rat1 | 660 | 928 | 135 | 79 | 54 | 100 | 54 | 89 | 1245 | 1645 |
| 12-month-old host_Rat2 | 152 | 1177 | 125 | 174 | 32 | 98 | 25 | 74 | 567 | 2038 |
| 12-month-old host_Rat3 | 1273 | 889 | 151 | 104 | 73 | 69 | 72 | 55 | 2168 | 1525 |

**Supplementary table 2.** Total cells analyzed for phenotypic identification in both BLT and Host cortex region of interests (ROI). N = 3 rats for each group.
