# Supplementary material for: Age-dependent integration of cortical progenitors transplanted at CSF-neurogenic niche interface
Source: Front Cell Dev Biol. 2025 Jul 3;13:1577045. doi: 10.3389/fcell.2025.1577045 (PMC12267260; doi:10.3389/fcell.2025.1577045)
Supplement: Supplementary file 4 [file DataSheet1.pdf]

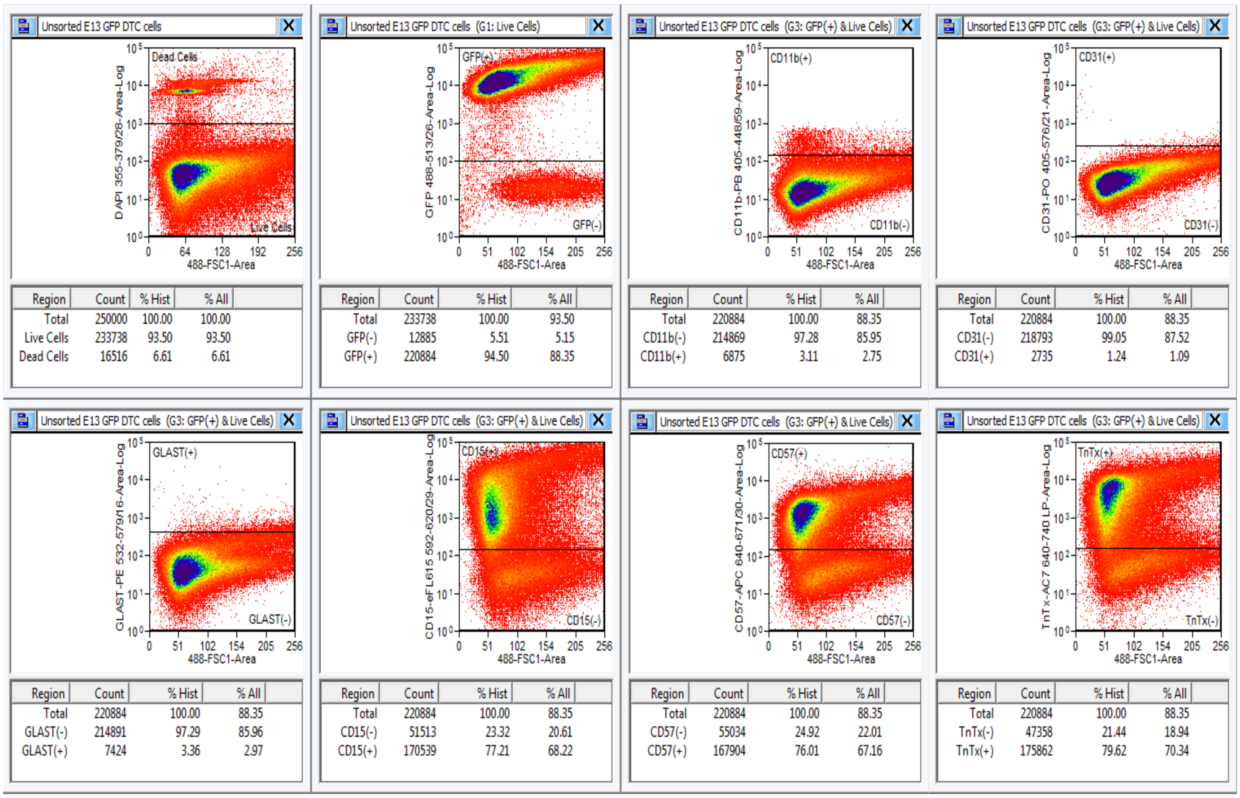

Supplementary Figure 1. FACS isolation of GFP-expressing lineage-negative E13.5 cortical neural precursor cells using different surface markers to deplete microglia (CD11b), endothelial cells (CD31), radial glia and astroglial progenitors (GLAST), early neuroglial progenitors (CD15), and differentiating post-mitotic neurons (CD57, TnTx). Live and GFP-positive cells were selected using FACS. Of those, cells that were positive for surface markers were removed from the pool.

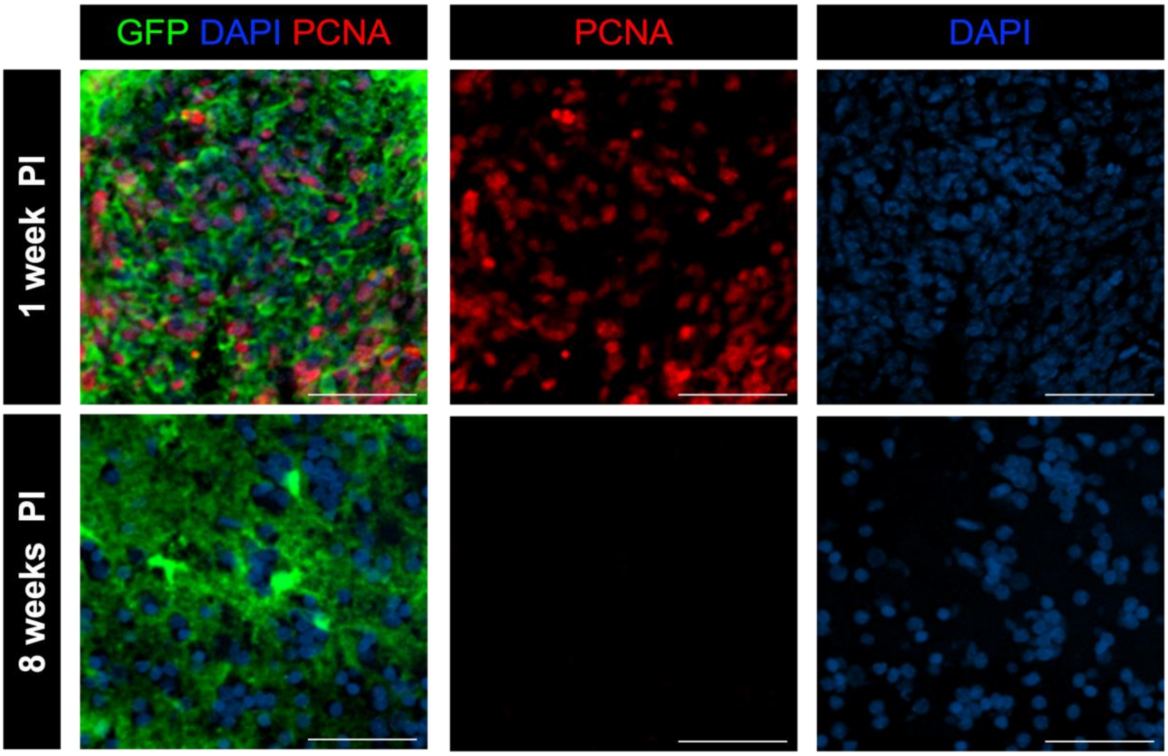

Supplementary Figure 2. Immunostaining with PCNA shows that initially high proliferation activity within the precursor-derived BLT observed at 1-week post-implantation subsided by approximately 8 weeks post-implantation. Scale bar = 100 μm.

A

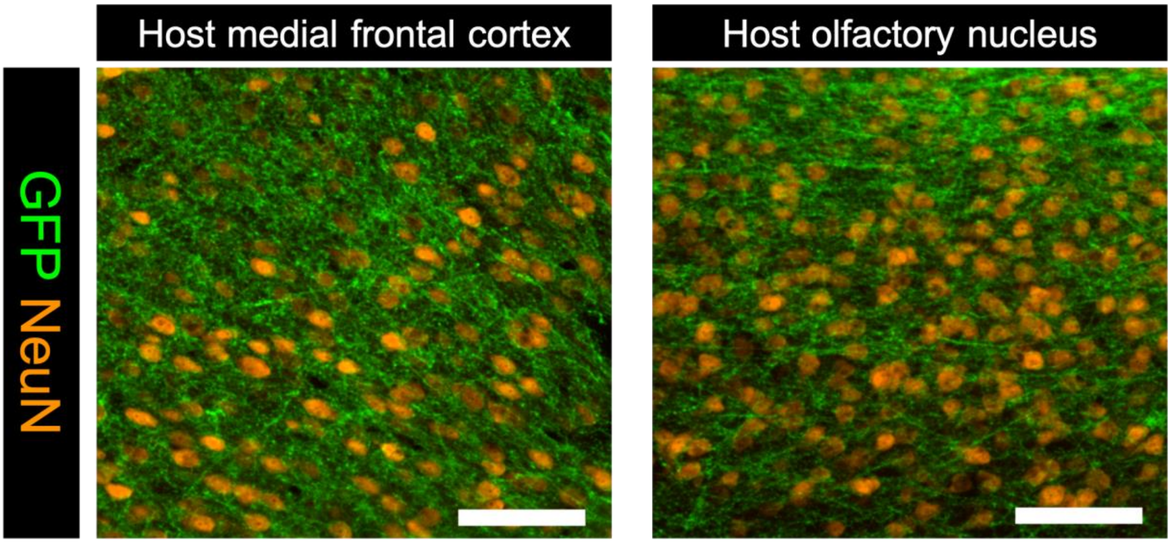

B

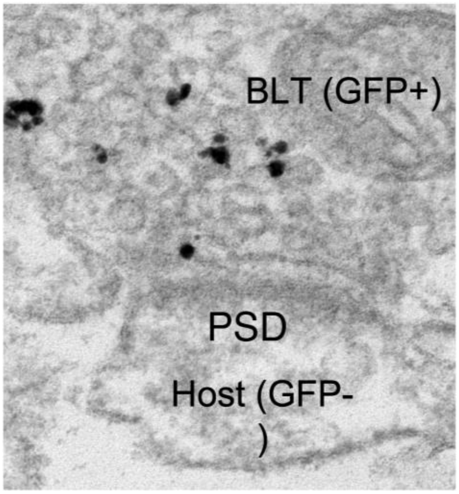

Supplementary Figure 3. A) High-magnification fluorescent images of neural processes from the GFP-positive BLT innervating the host neurons (NeuN-positive cells) in the host front cortex and host olfactory nucleus. Scale bar = 100 um. **B**) Electron micrograph of the presynaptic terminal from BLT (GFP-postive) that forms a synapse with the host (GFP-negative) neuron. A prominent postsynaptic dendity (PSD) indicates the excitatory nature of this synapse.

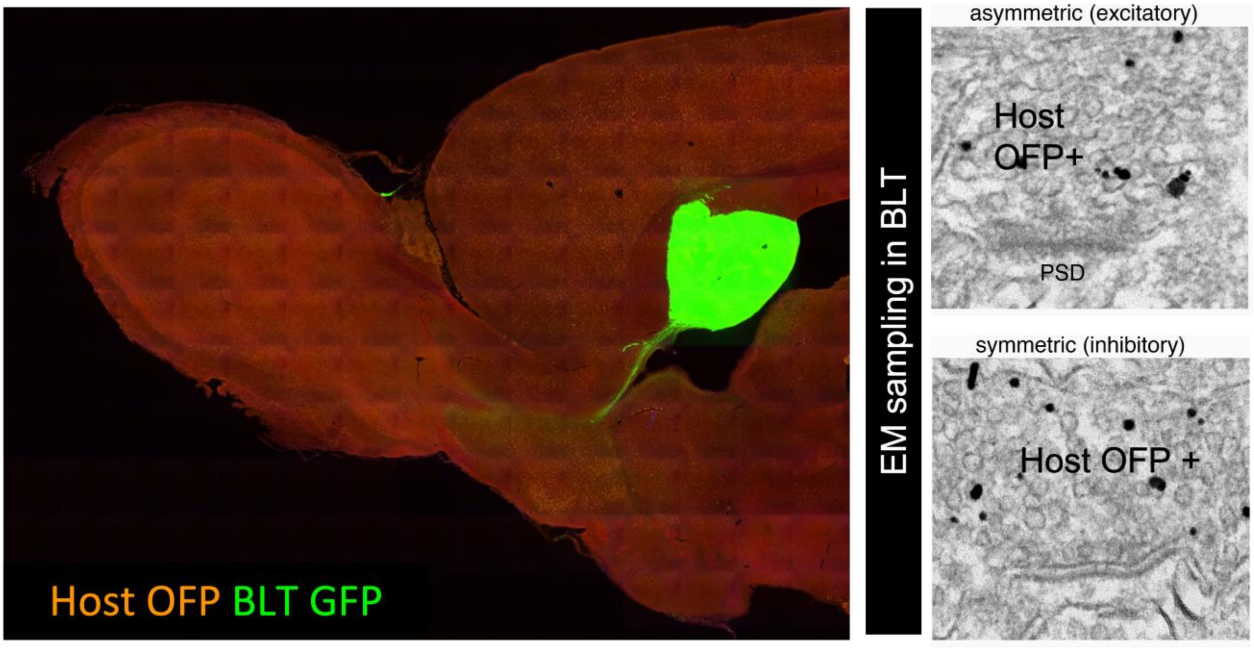

Supplementary Figure 4. Implantation of GFP-expressed precursor cells into OFP-expressed recipient. A grafted BLT (green, GFP-positive) integrating into a host brain (orange, OFP-positive). Electron micrographs showing asymmetric (excitatory) and symmetric (inhibitory) synapses from different OFP-positive host axons innervating onto the OFP-negative BLT denritic spine and shaft, respectively

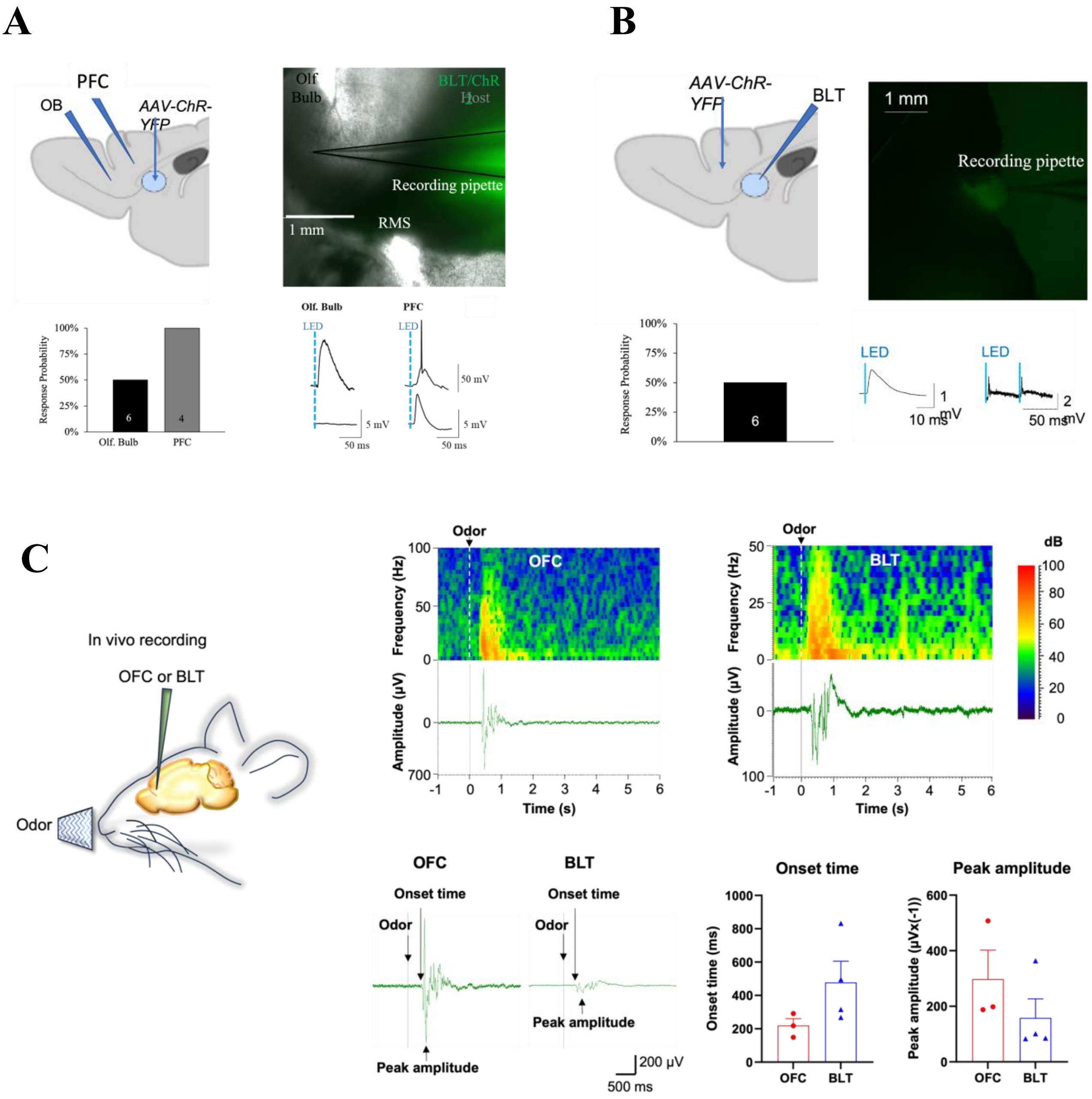

Supplementary Figure 5. Assessment of Functional Connectivity. **A**) Slice electrophysiology detects functional, monosynaptic connections from the ChR2-expressing BLT and the host prefrontal cortex and olfactory bulb. **B)** Slice electrophysiology shows synaptic connection from the ChR2-expressing host neurons in medial prefrontal cortex to the BLT. Note that some connections were strong and yielded action potentials (in PFC), while others were weaker and only produced depolarizations (olfactory bulb). **C)** In vivo recording of rat with BLT was completed using 10% amyl acetate diluted in mineral oil as stimulus. The left panel shows a schematic diagram of in vivo extracellular recordings using a 32-channel silicon probe. The top right panel shows LFP power spectrogram during odor stimulation in the PFC (left) and BLT (right). The bottom shows example raw traces of LFP signals. Black arrows indicate onset time and peak amplitude. Onset time was compared between the PFC group and the BLT group was not significant (PFC: N = 3, BLT: N = 4), t-test, $p > 0.05$. Mean ± SEM. Peak amplitude was compared between the two groups and no significance was found (PFC: N = 3, BLT: N = 4). t-test, $p > 0.05$. Mean ± SEM.

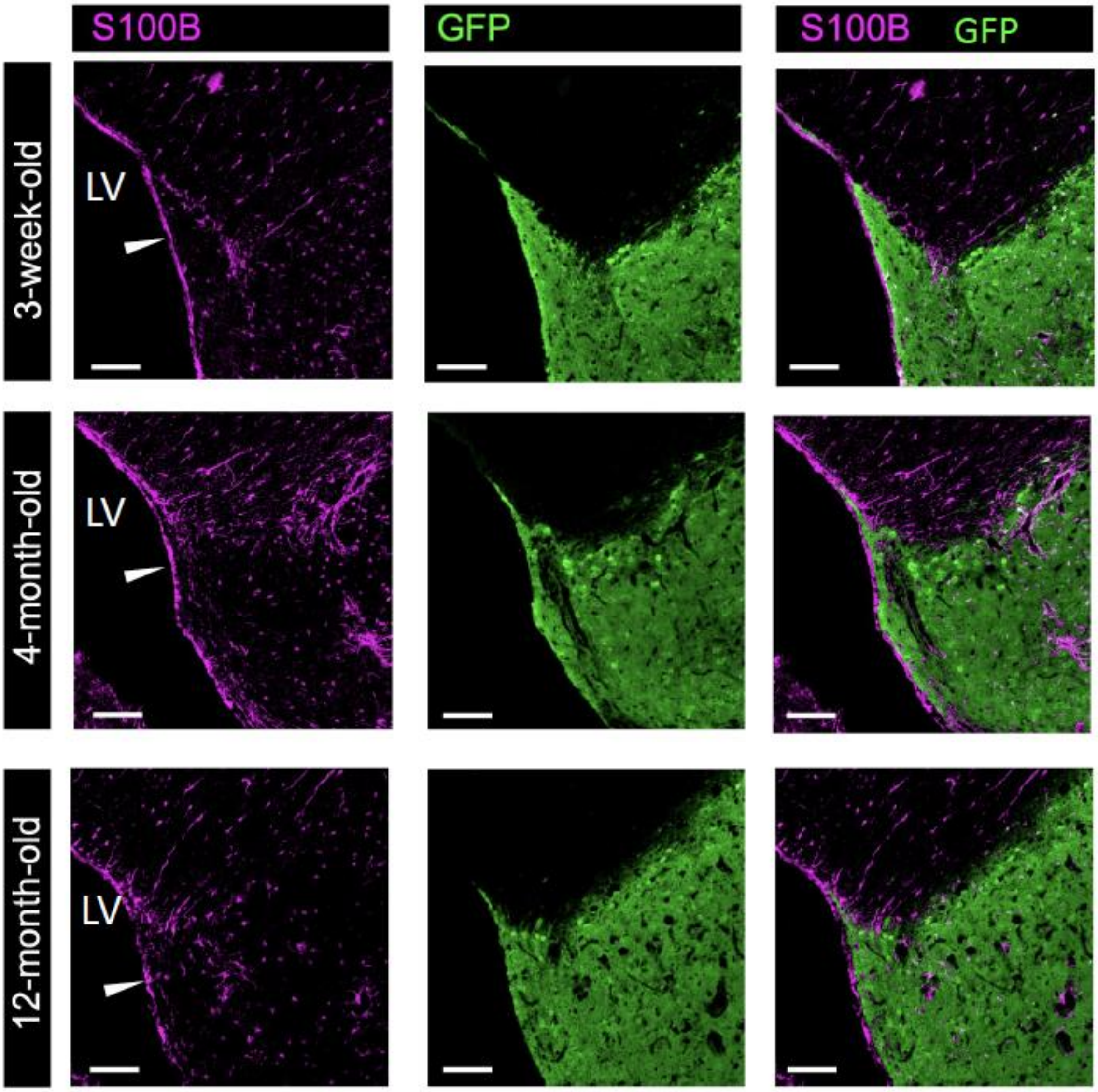

Supplementary Figure 6. Immunostaining with S100B shows host ependymal cell layer (arrowhead) extending from the host brain parenchyma partially encapsulate the transplant GFP-positive tissue and separate this tissue from direct contact with the CSF-filled lateral ventricle (LV). Scale bar = 100 μm.

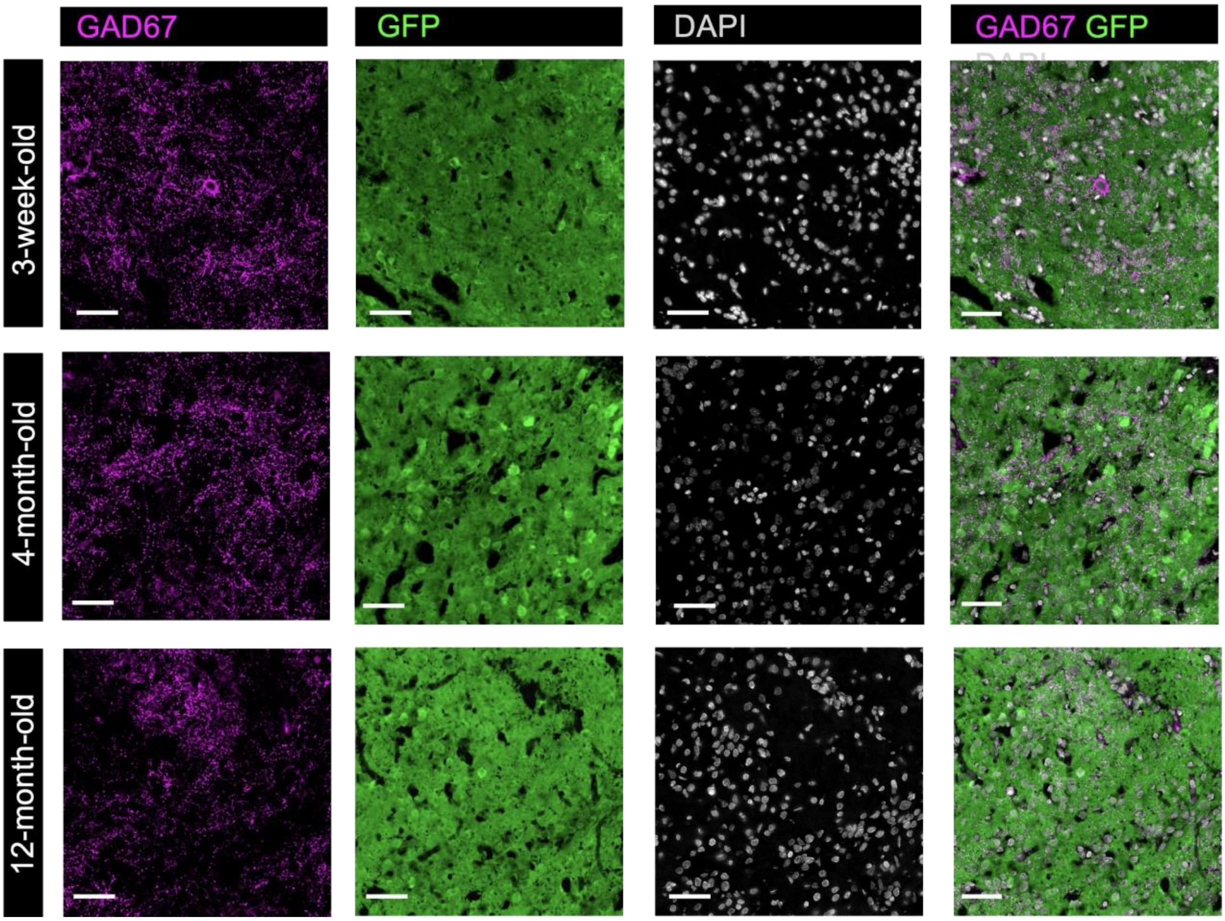

Supplementary Figure 7. Presence of GAD67 (inhibitory input) positive processes and neurons within the BLT transplants. Immunostaining with GAD67 (magenta) show that these cells and processes are GFP-negative. Therefore, these cells have originated exclusively from the host. Scale bar = 50 µm.

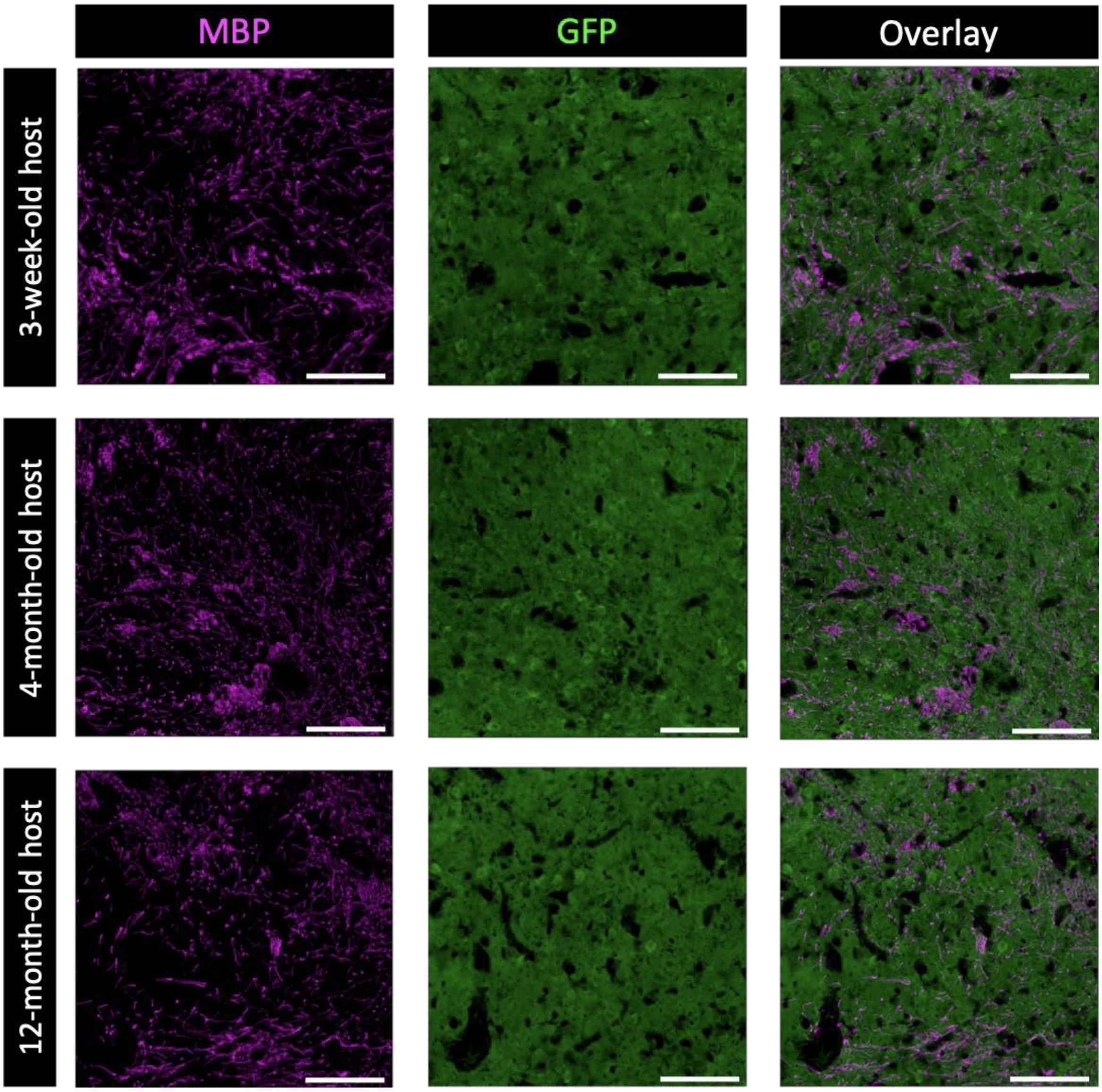

Supplementary Figure 8. Immunostaining with myelin basic protein (MBP) indicates extensive myelination of BLT transplants across the host ages. MBP processes are also GFP-negative and thus have originated from the host. Scale bar = 100 µm.
